# Basal protein phosphatase 2A activity restrains cytokine expression: role for MAPKs and tristetraprolin

**DOI:** 10.1038/srep10063

**Published:** 2015-05-18

**Authors:** Md. Mostafizur Rahman, Nowshin N. Rumzhum, Jonathan C. Morris, Andrew R. Clark, Nicole M. Verrills, Alaina J. Ammit

**Affiliations:** 1 Faculty of Pharmacy University of Sydney. NSW 2006 Australia; 2 School of Chemistry University of NSW. NSW 2052 Australia; 3 Centre for Translational Inflammation Research School of Immunity and Infection University of Birmingham. Edgbaston B15 2TT United Kingdom; 4 School of Biomedical Sciences and Pharmacy Faculty of Health University of Newcastle. NSW 2308 Australia

## Abstract

PP2A is a master controller of multiple inflammatory signaling pathways. It is a target in asthma; however the molecular mechanisms by which PP2A controls inflammation warrant further investigation. In A549 lung epithelial cells *in vitro* we show that inhibition of basal PP2A activity by okadaic acid (OA) releases restraint on MAPKs and thereby increases MAPK-mediated pro-asthmatic cytokines, including IL-6 and IL-8. Notably, PP2A inhibition also impacts on the anti-inflammatory protein – tristetraprolin (TTP), a destabilizing RNA binding protein regulated at multiple levels by p38 MAPK. Although PP2A inhibition increases TTP mRNA expression, resultant TTP protein builds up in the hyperphosphorylated inactive form. Thus, when PP2A activity is repressed, pro-inflammatory cytokines increase and anti-inflammatory proteins are rendered inactive. Importantly, these effects can be reversed by the PP2A activators FTY720 and AAL(s), or more specifically by overexpression of the PP2A catalytic subunit (PP2A-C). Moreover, PP2A plays an important role in cytokine expression in cells stimulated with TNFα; as inhibition of PP2A with OA or PP2A-C siRNA results in significant increases in cytokine production. Collectively, these data reveal the molecular mechanisms of PP2A regulation and highlight the potential of boosting the power of endogenous phosphatases as novel anti-inflammatory strategies to combat asthmatic inflammation.

Asthma is a clinically and socioeconomically significant disease driven by inflammation. Corticosteroids are the mainstay of anti-inflammatory therapy in respiratory disease and although they have proven clinical efficacy in asthma, many asthmatic inflammatory conditions (e.g. infection, exacerbation) are not responsive to them. Corticosteroid insensitivity can range from relative corticosteroid insensitivity to steroid resistance, as seen in severe asthma (reviewed in^1^,^2^). Thus, alternative anti-inflammatory strategies are urgently needed and enhancing the function of endogenous phosphatases, especially protein phosphatase 2A (PP2A), offer great promise.

PP2A is a master controller of multiple inflammatory signaling pathways. PP2A is a ubiquitously expressed serine/threonine phosphatase that exists as a tri-molecular complex of a catalytic subunit (C), a structural subunit (A), and a variable regulatory subunit (B) of which there are at least 3 different families (B55, B56, B”) each with several isoforms^3^. PP2A has generated much excitement as a target for anti-cancer therapy (reviewed in^4^} and has more recently emerged as a druggable target in respiratory disease^5^,^6^. But in order to accelerate the development of PP2A activators as a future pharmacotherapeutic strategy in respiratory medicine, it is essential that we gain an advanced understanding of the regulation and function of PP2A in cellular models of asthmatic inflammation *in vitro*.

PP2A dephosphorylates a number of kinases that drive inflammatory cell signaling^7^,^8^. Notably, PP2A can dephosphorylate members of the mitogen-activated protein kinase (MAPK) superfamily, including p38 MAPK^8^. By regulating MAPKs, PP2A exerts significant control over cytokine regulatory networks; although the molecular mechanisms responsible remain relatively unexplored in airway inflammation.

We address this herein by utilizing the human alveolar epithelial cell line (A549); a transformed cell line widely used to examine cytokine expression in the context of asthmatic inflammation^9^,^10^,^11^. Confirmatory experiments were also performed with the human bronchial epithelial cell line (BEAS-2B)^5^,^9^,^12^. PP2A is a ubiquitously expressed phosphatase and our study shows that under unstimulated conditions there is a high level of PP2A enzymatic activity. This basal PP2A activity serves to restrain downstream effectors regulated by MAPKs. Inhibition of PP2A releases restraint and thereby increases MAPK-mediated pro-inflammatory cytokines, including interleukin 6 and 8 (IL-6 and IL-8), as well as disable the anti-inflammatory function of tristetraprolin (TTP), a destabilizing RNA binding protein regulated at multiple levels by p38 MAPK.

## Methods

### Chemicals

Okadaic acid (OA) was purchased from Enzo Life Sciences (Farmingdale, NY). FTY720 was purchased from Cayman Chemical Company (Ann Arbor, MI) and AAL(s) was synthesized^5^,^6^,^13^. Tumor necrosis factor α (TNFα) is from R&D Systems (Minneapolis, MN). Unless otherwise specified, all chemicals used in this study were purchased from Sigma-Aldrich (St. Louis, MO).

### Cell culture

The human alveolar epithelial cell line (A549) and human bronchial epithelial cell line (BEAS-2B) were cultured in Ham’s F-12K (Kaighn’s) Medium (Invitrogen, Carlsbad, CA) supplemented with penicillin (100 U/ml), streptomycin (100 μg/ml), and fetal calf serum (10%), in accordance with culture conditions reported by Cornell *et al.*^9^. All experiments were performed after an overnight serum-starvation period (14-16 h) in Ham’s F-12K supplemented with sterile BSA (0.1%). A minimum of three experimental replicates performed on separate days were used for each experiment.

### PP2A activity assay

PP2A activity was determined using the PP2A immunoprecipitation phosphatase assay kit (Merck Millipore, Darmstadt, Germany) according to the manufacturer’s instructions.

### Western blotting

Western blotting was performed using rabbit monoclonal or polyclonal antibodies against phosphorylated (Thr^180^/Tyr^182^) and total p38 MAPK, phosphorylated (Thr^202^/Tyr^204^) and total ERK, phosphorylated (Thr^183^/Tyr^185^) and total JNK (all from Cell Signaling Technology, Danvers, MA). The catalytic subunit of PP2A (PP2A-C) was detected with a mouse monoclonal antibody (IgG2bκ, clone 1D6: Merck Millipore, Darmstadt, Germany). TTP was measured by Western blotting using rabbit antisera against TTP (Sak21). Detection of α-tubulin was used as the loading control (mouse monoclonal IgG_1_, DM1A: Santa Cruz Biotechnology, Santa Cruz, CA). Primary antibodies were detected with goat anti-rabbit and anti-mouse HRP-conjugated secondary antibodies (Cell Signaling Technology, Danvers, MA) and visualized by enhanced chemiluminescence (PerkinElmer, Wellesley, MA).

### Real-time RT-PCR

Total RNA was extracted using the RNeasy Mini Kit (Qiagen Australia, Doncaster, VIC, Australia) and reverse transcription performed by using the RevertAid First strand cDNA Synthesis Kit (Fermentas Life Sciences, Hanover, MD) according to the manufacturer’s protocol. IL-6, IL-8 and TTP mRNA levels were measured using real-time RT-PCR on an ABI Prism 7500 (Applied Biosystems, Foster City, CA) with IL-6 (Hs00174131_m1), IL-8 (Hs00174103_m1) and TTP (Zfp36, Hs00185658_m1) TaqMan gene expression assays and the eukaryotic 18S rRNA endogenous control probe (Applied Biosystems) subjected to the following cycle parameters: 50 °C for 2 min, 1 cycle; 95 °C for 10 min, 1 cycle; 95 °C for 15 s, 60 °C for 1 min, 40 cycles and mRNA expression (fold increase) quantified by delta delta Ct calculations.

### ELISAs

IL-6 and IL-8 ELISAs were performed according to the manufacturer’s instructions (BD Biosciences Pharmingen, San Diego, CA).

### Transient transfection

A549 cells (5 × 10^5^ cells/well) were transfected with 1 μg of pEGFP HA-PP2A-C, or empty vector control, using Lipofectamine 2000 (Invitrogen). After transfection, cells were cultured for 24 h in media without antibiotics, and then growth-arrested for 16 h in Ham’s F-12K supplemented with 0.1% BSA, supplemented with penicillin (100 U/ml), streptomycin (100 μg/ml), before cells were assayed.

### siRNA transfection

A549 cells (5 × 10^5^ cells/well) were transfected with siRNA against PP2A-C, or scrambled control, by reverse transfection with RNAiMAX according to the manufacturer’s protocols (Invitrogen, NY, USA). Specifically, for each well of 6-well plates, 800 ng of ON-Target plus Control Non-targeting siRNA (scrambled control) or ON-target plus SMART pool Human PPP2CA siRNA (aka PP2A-C: both from Dharmacon, Thermo-Fisher Scientific, Waltham, MA) was diluted in 500 μL of Opti-MEM Reduced Serum Media (Invitrogen). This was followed by the addition of 5 μL of RNAiMAX reagent (Invitrogen) into each well and incubation at room temperature for 20 min. After transfection, cells were cultured for 24 h in media without antibiotics, and then growth-arrested for 16 h in Ham’s F-12K supplemented with 0.1% BSA, supplemented with penicillin (100 U/ml), streptomycin (100 μg/ml), before stimulation with TNFα (4 ng/ml).

### Statistical analysis

Statistical analysis was performed using either the Student’s unpaired *t* test, one-way or two-way ANOVA followed by Bonferroni’s post-test. *P* values < 0.05 were sufficient to reject the null hypothesis for all analyses. Data are mean ± SEM of n ≥ 3 independent replicates.

## Results

### Temporal regulation of basal PP2A enzymatic activity by OA

**Figure 1:**
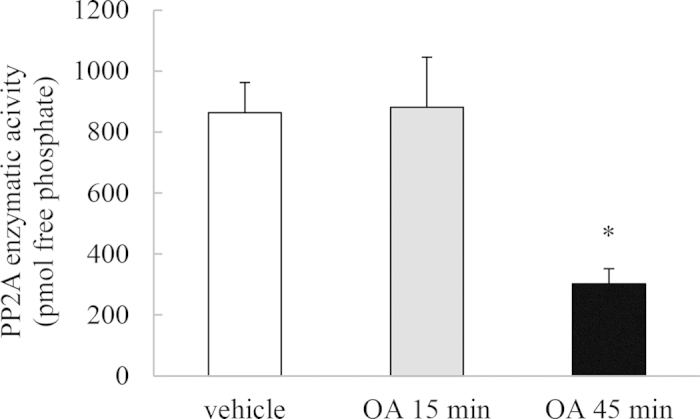
Temporal regulation of basal PP2A enzymatic activity by OA. PP2A enzymatic activity was measured in A549 cells treated for 15 min or 45 min with 1 μM OA, compared to vehicle-treated cells. Cells were washed and then PP2A enzymatic activity (measured as pmol free phosphate) detected at 1 h. Statistical analysis was performed using the Student’s unpaired *t* test where * denotes a significant decrease in PP2A activity compared to vehicle-treated cells (*P* < 0.05). Data are mean + SEM values from n = 3 independent experiments.

OA is a non-selective pharmacological inhibitor of PP2A^9^,^14^ and widely used to potently inhibit PP2A phosphatase activity^9^,^15^,^16^,^17^. To examine the temporal regulation of basal PP2A enzymatic activity by OA, A549 cells were treated with 1 μM OA for 15 min or 45 min, then washed and left for a further 1 h before measuring PP2A enzymatic activity. PP2A is a ubiquitously expressed phosphatase and, as shown in Fig. 1, the basal PP2A enzymatic activity in A549 cells is 863.7 ± 98.9 pmol free phosphate. This activity can be significantly repressed by 45 min treatment with OA, while treatment for a shorter time period (i.e. 15 min) was without effect. These data indicate in part, the temporal regulation of basal PP2A activity by OA. Results from cells treated for both time points will be included throughout the study to demonstrate the link between repression of basal PP2A activity and effects on functional outcomes such as cell signaling and cytokine expression.

### Inhibition of basal PP2A phosphatase activity allows unrestrained action of MAPK phosphoproteins

**Figure 2:**
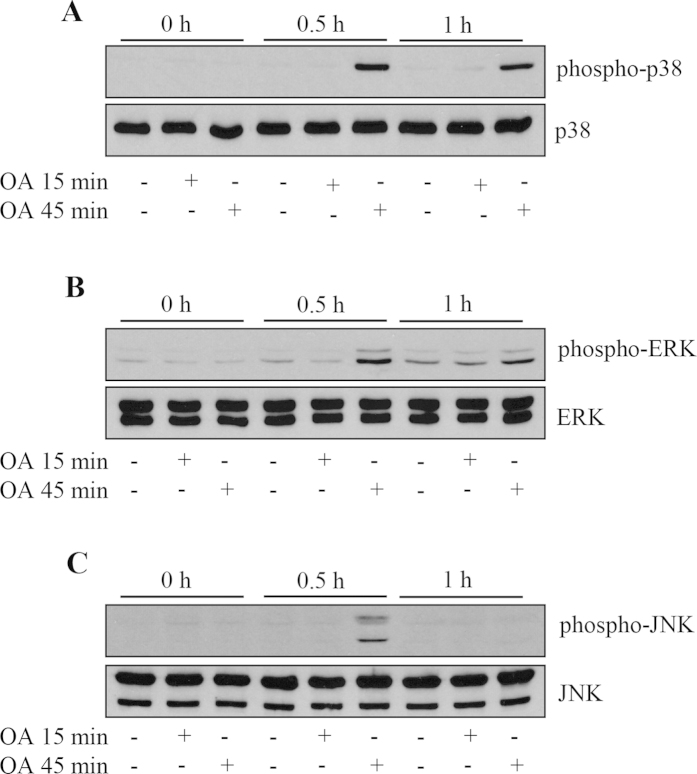
Inhibition of basal PP2A phosphatase activity allows unrestrained action of MAPK phosphoproteins. A549 cells were treated for 15 min or 45 min with 1 μM OA, compared to vehicle. Cells were washed and then lysates prepared at 0, 0.5 h, and 1 h, to compare temporal kinetics of (**A**) p38 MAPK, (**B**) ERK and (**C**) JNK phosphorylation by Western blotting (representative results (from n = 5 independent experiments) are shown as cropped blots and full-length blots are presented in Supplementary Figure 1A (p38 MAPK), 1B (ERK) and 1C (JNK)).

PP2A dephosphorylates a number of kinases that drive inflammatory cell signaling; thus its inhibition allows unrestrained action of a number of downstream effectors. MAPKs family members (p38 MAPK, ERK and JNK) are important regulators of cytokine expression and are known to drive expression of two important cytokines implicated in asthmatic inflammation, IL-6 and IL-8 18,19,20,21. Accordingly, we examined the effect of OA on p38 MAPK, ERK and JNK phosphorylation by Western blotting. As shown in Fig. 2A, treating cells for 45 min with OA robustly increased p38 MAPK phosphorylation at 0.5 and 1 h. ERK phosphorylation was enhanced at 30 min, and to a lesser extent at 1 h (Fig. 2B). JNK phosphorylation was also enhanced at 30 min under these conditions (Fig. 2C). Cells treated for the shorter time period of 15 min with OA did not show enhanced activity of MAPK phosphoproteins.

### Treating A549 cells with OA for 45 min, but not 15 min, significantly increases IL-6 mRNA expression and protein secretion

**Figure 3:**
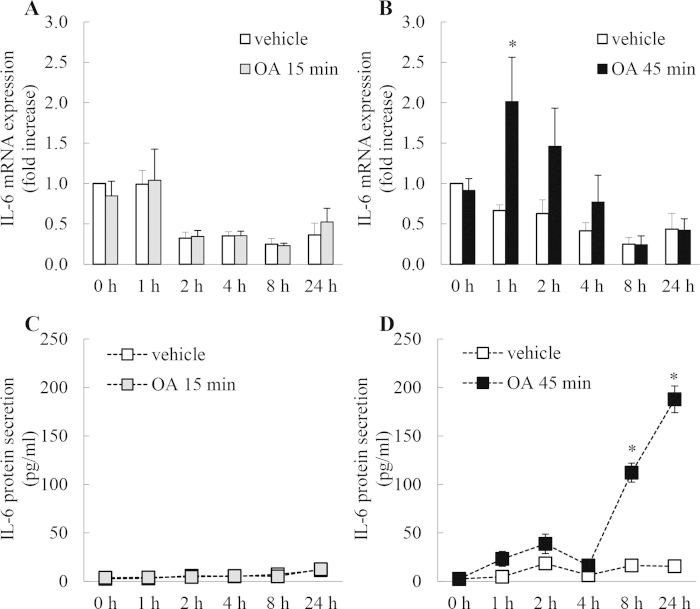
Treating A549 cells with OA for 45 min, but not 15 min, significantly increases IL-6 mRNA expression and protein secretion. A549 cells were treated for (**A**, **C**) 15 min or (**B**, **D**) 45 min with 1 μM OA, compared to vehicle. Cells were washed and then (**A**, **B**) IL-6 mRNA expression (results expressed as fold increase compared to vehicle-treated cells at 0 h) and (**C**, **D**) IL-6 protein secretion measured at 0, 1, 2, 4, 8, and 24 h. Statistical analysis was performed using two-way ANOVA then Bonferroni’s post-test (where * denotes a significant effect compared to vehicle-treated cells (*P* < 0.05)). Data are mean ± SEM values from n = 4 independent experiments.

We then examined whether the inhibition of basal PP2A with OA has an effect on IL-6 mRNA expression and protein secretion. As shown in Fig. 3B, 45 min treatment with OA induced significant upregulation of IL-6 mRNA expression in a temporally distinct manner, with the peak of expression observed at 1 h (Fig. 3B: *P* < 0.05). This resulted in significant increase in IL-6 secretion observed at 8 and 24 h (Fig. 3D: *P* < 0.05). IL-6 production is p38 MAPK-mediated 18,20, thus, taken together with our earlier results, these data indicate that significant repression of basal PP2A activity by 45 min treatment with OA allows p38 MAPK activation and corresponding increases in p38 MAPK-mediated cytokines such as IL-6. This PP2A-dependency of these results is confirmed by the lack of IL-6 mRNA expression and protein secretion in cells where basal PP2A activity was unaffected (see negative results in cells treated for only 15 min with OA (Fig. 3A,C)).

### Treating A549 cells with OA for 45 min, but not 15 min, significantly increases IL-8 mRNA expression and protein secretion

**Figure 4:**
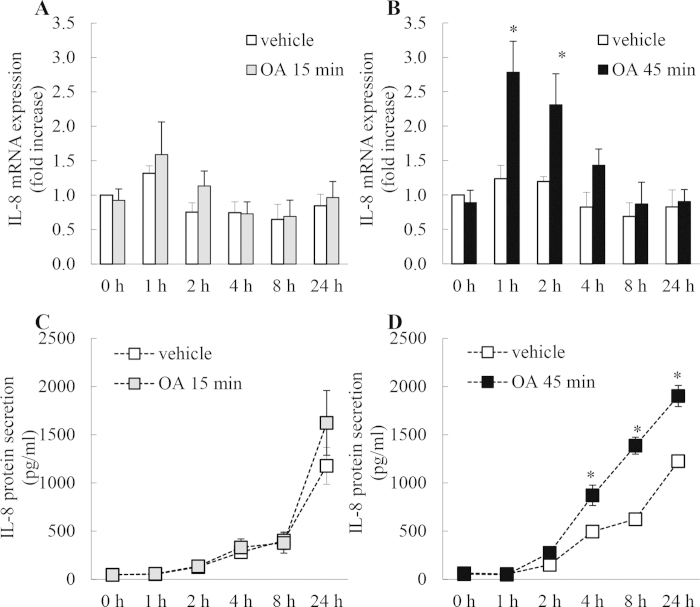
Treating A549 cells with OA for 45 min, but not 15 min, significantly increases IL-8 mRNA expression and protein secretion. A549 cells were treated for (**A**, **C**) 15 min or (**B**, **D**) 45 min with 1 μM OA, compared to vehicle. Cells were washed and then (**A**, **B**) IL-8 mRNA expression (results expressed as fold increase compared to vehicle-treated cells at 0 h) and (**C**, **D**) IL-8 protein secretion measured at 0, 1, 2, 4, 8, and 24 h. Statistical analysis was performed using two-way ANOVA then Bonferroni’s post-test (where * denotes a significant effect compared to vehicle-treated cells (*P* < 0.05)). Data are mean ± SEM values from n = 4 independent experiments.

We then examined the effect of OA on IL-8 mRNA expression and protein secretion. Figure 4A reveals that 15 min treatment with OA has no effect on IL-8 mRNA expression but 45 min treatment significantly increased IL-8 mRNA at 1 h and 2 h time point (Fig. 4B: *P* < 0.05). Similarly, treatment with OA for 15 min did not increase IL-8 secretion above that achieved in cells treated with vehicle alone (Fig. 4C), while significant increases in IL-8 secretion were observed at 4, 8, and 24 h after 45 min treatment with OA, compared to vehicle-treated cells (Fig. 4D).

### Treating human bronchial epithelial cells (BEAS-2B) with OA for 45 min, but not 15 min, decreases PP2A enzymatic activity and significantly increases IL-6 and IL-8 mRNA expression and protein secretion

**Figure 5:**
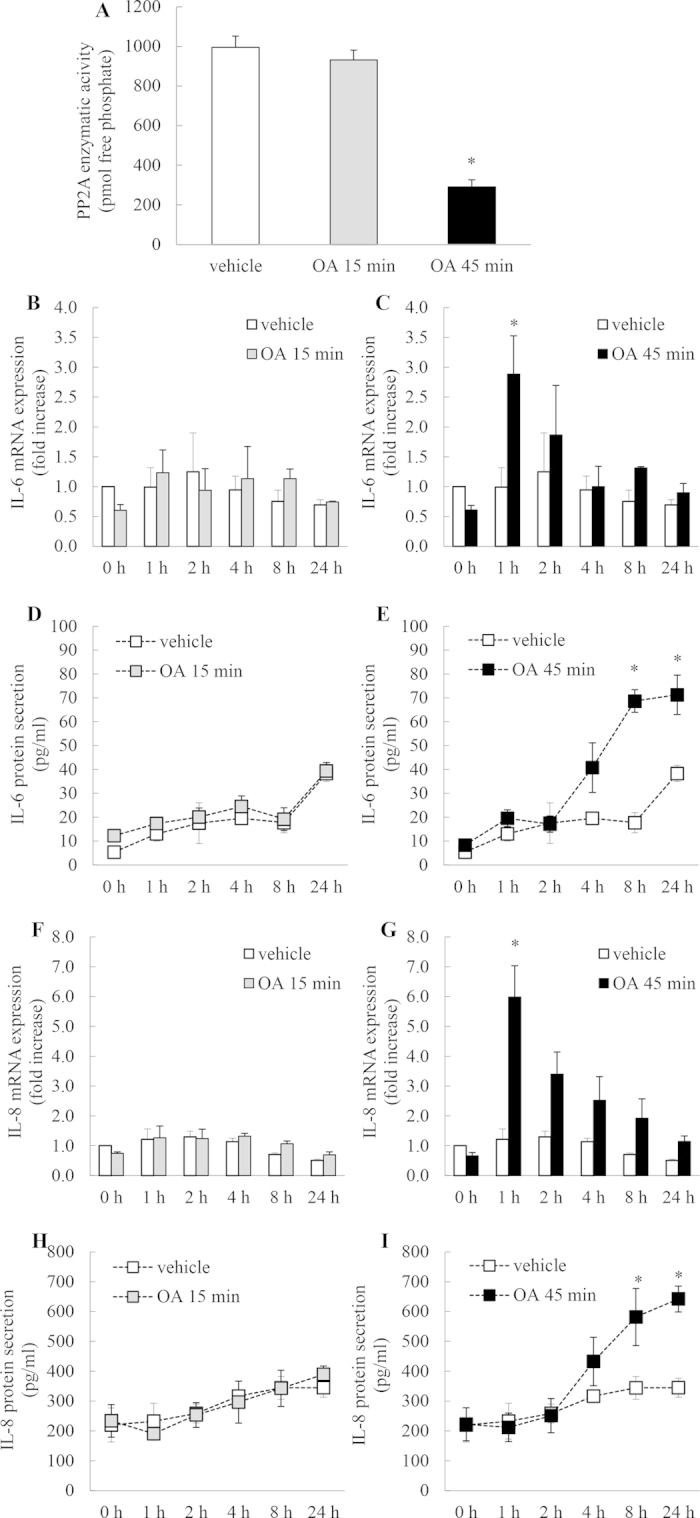
Treating human bronchial epithelial cells (BEAS-2B) with OA for 45 min, but not 15 min, decreases PP2A enzymatic activity and significantly increases IL-6 and IL-8 mRNA expression and protein secretion. (**A**) PP2A enzymatic activity was measured in BEAS-2B cells treated for 15 min or 45 min with 1 μM OA, compared to vehicle-treated cells. Cells were washed and then PP2A enzymatic activity (measured as pmol free phosphate) detected at 1 h. Statistical analysis was performed using the Student’s unpaired *t* test where * denotes a significant decrease in PP2A activity compared to vehicle-treated cells (*P* < 0.05). BEAS-2B cells were treated for (**B**, **D**, **F**, **H**) 15 min or (**C**, **E**, **G**, **I**) 45 min with 1 μM OA, compared to vehicle. Cells were washed and then (**B**, **C**) IL-6 and (**F**, **G**) IL-8 mRNA expression (results expressed as fold increase compared to vehicle-treated cells at 0 h) and (**D**, **E**) IL-6 and (**H**, **I**) IL-8 protein secretion measured at 0, 1, 2, 4, 8, and 24 h. Statistical analysis was performed using two-way ANOVA then Bonferroni’s post-test (where * denotes a significant effect compared to vehicle-treated cells (*P* < 0.05)). Data are mean ± SEM values from n = 3 independent experiments.

In order to confirm these findings in a second immortalized cell line, we utilized the human epithelial cell line BEAS-2B as they commonly used in *in vitro* studies examining airway inflammation with relevance to asthma^5^,^9^,^12^,^22^,^23^,^24^. We conducted a series of confirmatory experiments (Fig. 5) to demonstrate that treating BEAS-2B cells with OA for 45 min, but not 15 min, decreases PP2A enzymatic activity (Fig. 5A) and significantly increases IL-6 mRNA expression (Fig. 5C) and IL-6 secretion (Fig. 5E) (P < 0.05). IL-8 mRNA expression and protein secretion was also affected by 45 min treatment with OA (Fig. 5G,I, respectively: *P* < 0.05). These results confirm observations observed in A549 cells (Figs. 3 and 4).

### Treating A549 cells with OA for 45 min, but not 15 min, significantly increases TTP mRNA expression and upregulation of TTP protein that is hyperphosphorylated

TTP is an important anti-inflammatory protein that is a direct target of PP2A^25^ and can be regulated at multiple levels by p38 MAPK. TTP is an immediate early response gene whose expression is p38 MAPK-dependent^26^ and once expressed its protein stability is regulated post-translationally by p38 MAPK-mediated phosphorylation of two key serines^10^,^25^,^26^. Importantly, this latter step also controls TTP function as an RNA destabilizing protein (phosphorylated – OFF; unphosphorylated – ON). Because PP2A controls TTP, we were interested to examine the impact of repression of PP2A activity on this TTP expression and function.

**Figure 6:**
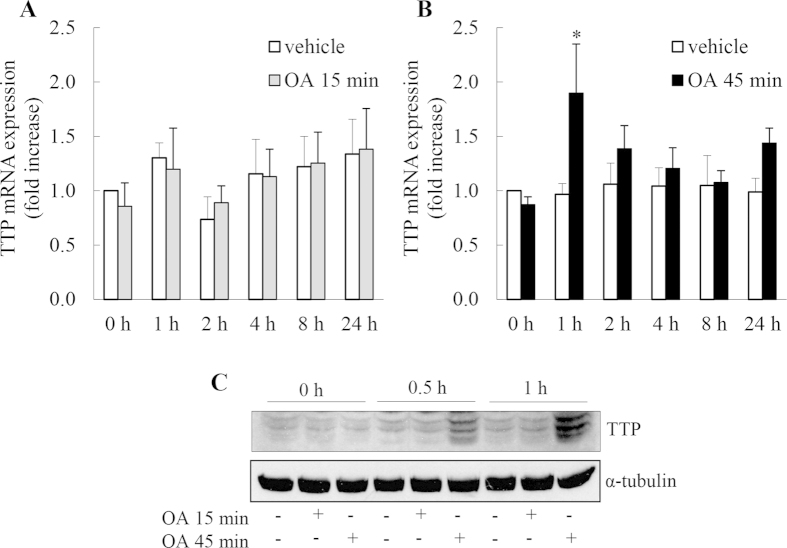
Treating A549 cells with OA for 45 min, but not 15 min, significantly increases TTP mRNA expression and upregulation of TTP protein that is hyperphosphorylated. A549 cells were treated for 15 min or 45 min with 1 μM OA, compared to vehicle. Cells were washed and at the indicated times (**A**, **B**) TTP mRNA expression (results expressed as fold increase compared to vehicle-treated cells at 0 h) and (**C**) TTP protein upregulation was measured by Western blotting with α-tubulin as the loading control (representative results are shown as cropped blots and full-length blots are presented in Supplementary Figure 2). Please note that bands of immunoreactivity for TTP at higher molecular weight indicate phosphorylated forms of TTP. Statistical analysis was performed using two-way ANOVA then Bonferroni’s post-test (where * denotes a significant effect compared to vehicle-treated cells (*P* < 0.05)). Data are mean + SEM values from n = 4 independent experiments.

Firstly, we examined TTP mRNA expression, and as shown in Fig. 6A, 15 min treatment with OA had no effect on the time course of TTP mRNA expression. In contrast, 45 min treatment with OA significantly increased TTP mRNA expression at 1 h (Fig. 6B: *P* < 0.05). Secondly, we measured TTP protein expression and phosphorylation with the rabbit antisera Sak21^10^,^26^. As shown in Fig. 6C, treating cells with OA for 45 min, but not for 15 min, increases TTP protein levels. Notably, we observe that the higher molecular weight immunoreactive bands for TTP predominate, especially at 1 h. This is consistent with earlier reports that inhibition of PP2A results in an equilibrium shift towards phosphorylated TTP, which is stable and builds up as a hyperphosphorylated (inactive) TTP^25^,^27^.

### The PP2A activator FTY720 overcomes OA-mediated inhibition of basal PP2A phosphatase activity and significantly represses IL-6 and IL-8 mRNA expression and cytokine secretion

**Figure 7:**
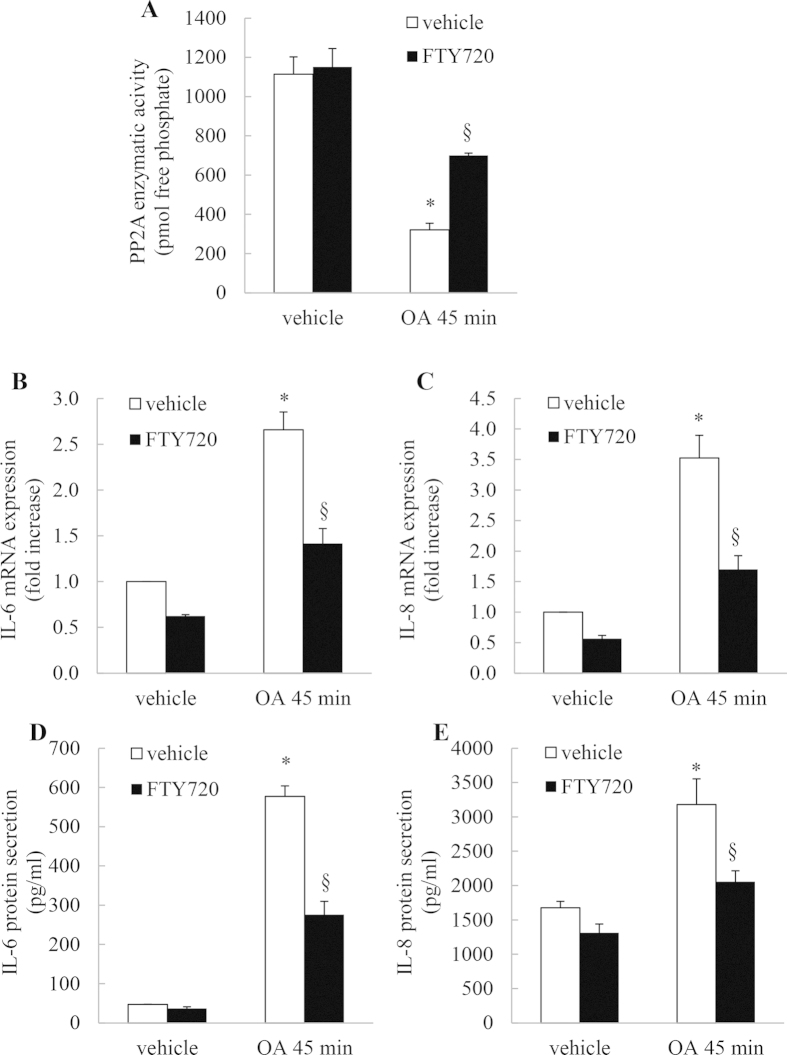
The PP2A activator FTY720 overcomes OA-mediated inhibition of basal PP2A phosphatase activity and significantly represses IL-6 and IL-8 mRNA expression and cytokine secretion. A549 cells were treated for 6 h with 2.5 μM FTY720 prior to 45 min with 1 μM OA, compared to vehicle. Cells were washed and then (**A**) PP2A activity measured at 1 h, (**B**, **C**) IL-6 and IL-8 mRNA expression measured at 1 h (results expressed as fold increase compared to vehicle-treated cells) and (**D**, **E**) IL-6 and IL-8 protein secretion measured at 24 h. Statistical analysis was performed using one-way ANOVA then Bonferroni’s post-test (where * denotes a significant effect of OA or § FTY720) (*P* < 0.05)). Data are mean + SEM values from n = 3 independent experiments.

Collectively, our data thus far implicates PP2A as a key regulator of cytokine expression via a MAPK/TTP-regulated network. That is, when PP2A is inhibited, cytokine secretion ensues. To demonstrate this further, we used a PP2A activator FTY720 (2-amino-2-[2-(4-octylphenyl)ethyl]-1,3-propanediol hydrochloride^28^) to overcome the effect of the PP2A inhibitor (OA). A549 cells were pretreated with FTY720 before treatment for 45 min with OA, or vehicle. Firstly, we quantitated PP2A enzymatic activity and found that in FTY720-treated cells, OA is unable to inhibit PP2A activity to the same extent (Fig. 7A: *P* < 0.05). Secondly, we measured IL-6 and IL-8 mRNA expression at 1 h and protein secretion at 24 h under these conditions. Cells pretreated with FTY720 had less significantly IL-6 (Fig. 7B) and IL-8 (Fig. 7C) mRNA expression than OA-treated controls. IL-6 and IL-8 protein secretion results also followed this pattern, as shown in Fig. 7D,E, respectively.

### The PP2A activator devoid of sphingosine 1- phosphate agonism - AAL(s) - overcomes OA-mediated inhibition of basal PP2A phosphatase activity and significantly represses IL-6 and IL-8 mRNA expression and cytokine secretion

**Figure 8:**
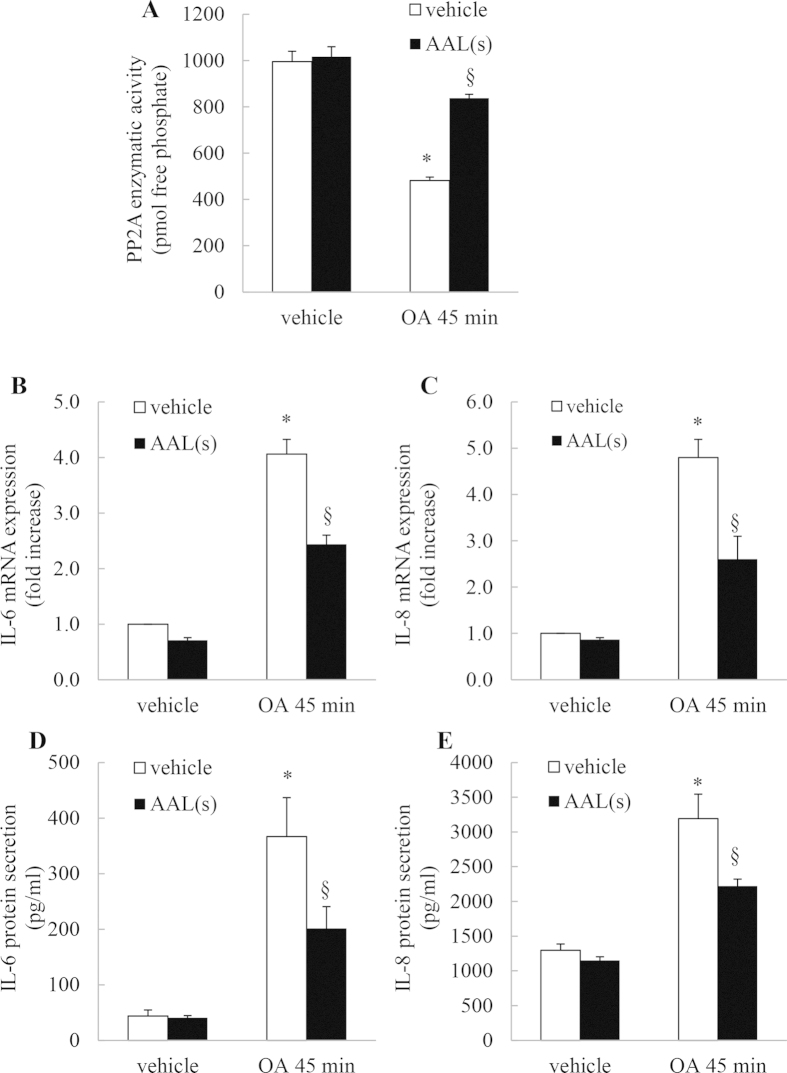
The PP2A activator devoid of sphingosine 1- phosphate agonism - AAL(s) -overcomes OA-mediated inhibition of basal PP2A phosphatase activity and significantly represses IL-6 and IL-8 mRNA expression and cytokine secretion. A549 cells were treated for 6 h with 2.5 μM AAL(s) prior to 45 min with 1 μM OA, compared to vehicle. Cells were washed and then (**A**) PP2A activity measured at 1 h, (**B**, **C**) IL-6 and IL-8 mRNA expression measured at 1 h (results expressed as fold increase compared to vehicle-treated cells) and (**D**, **E**) IL-6 and IL-8 protein secretion measured at 24 h. Statistical analysis was performed using one-way ANOVA then Bonferroni’s post-test (where * denotes a significant effect of OA or § AAL(s) (*P* < 0.05)). Data are mean + SEM values from n = 3 independent experiments.

Although FTY720 is a known activator of PP2A, it also has other targets. Most notably in the context of asthma, FTY720 can induce sphingosine 1-phosphate (S1P) signaling and we have previously shown that S1P is elevated in allergic asthma^29^, drives development of the pro-asthmatic phenotype, and can induce IL-6 and IL-8 expression^29^,^30^,^31^ . Although we did not observe upregulation of cytokine production with FTY720 in A549 cells, we still performed further experimentation with the FTY720 derivative 2-amino-4-(4-heptyloxyphenyl)-2-methylbutanol [AAL(s)]^5^,^6^,^13^ because AAL(s) is devoid of S1P agonism. The data shown in Fig. 8 serve to confirm that these effects are specific to PP2A by demonstrating that PP2A activation with AAL(s) can overcome OA-mediated inhibition of basal PP2A phosphatase activity and significantly repress IL-6 and IL-8 mRNA expression and cytokine secretion.

### Overexpression of the catalytic subunit of PP2A (PP2A-C) overcomes OA-mediated inhibition of basal PP2A phosphatase activity and significantly represses IL-6 and IL-8 mRNA expression and cytokine secretion

**Figure 9:**
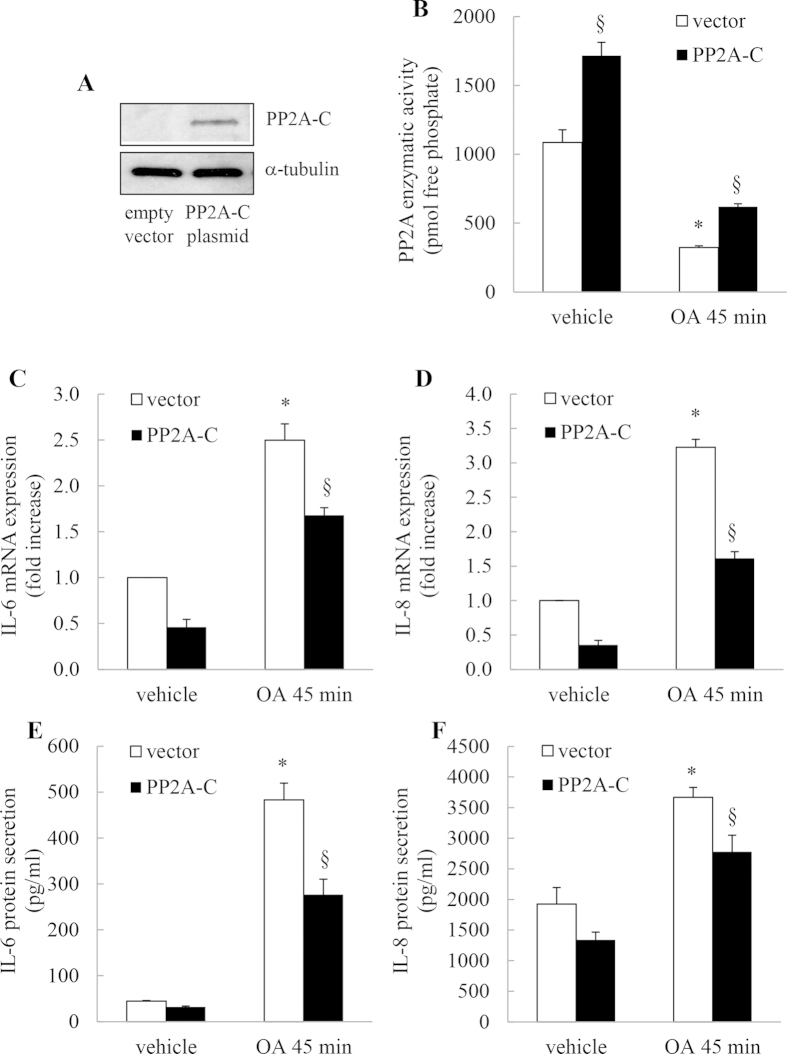
Overexpression of the catalytic subunit of PP2A (PP2A-C) overcomes OA-mediated inhibition of basal PP2A phosphatase activity and significantly represses IL-6 and IL-8 mRNA expression and cytokine secretion. A549 cells were transfected with empty vector or plasmid expressing PP2A-C, prior to 45 min with 1 μM OA, compared to vehicle. Cells were washed and then (**A**) PP2A-C overexpression confirmed by Western blotting (representative results are shown as cropped blots and full-length blots are presented in Supplementary Figure 3), (**B**) PP2A activity measured at 1 h, (**C**, **D**) IL-6 and IL-8 mRNA expression measured at 1 h (results expressed as fold increase compared to vehicle-treated cells) and (**E**, **F**) IL-6 and IL-8 protein secretion measured at 24 h. Statistical analysis was performed using one-way ANOVA then Bonferroni’s post-test (where * denotes a significant effect of OA or § PP2A-C) (*P* < 0.05)). Data are mean + SEM values from n = 3 independent experiments.

As further substantiation of the role of PP2A in the control of cytokine secretion we overexpressed the catalytic subunit of PP2A (PP2A-C) and measured the impact on IL-6 and IL-8 expression induced by OA. We first confirmed expression of PP2A-C in cells transfected with empty vector or PP2A-C (Fig. 9A) and then examined the impact of OA treatment (45 min) on PP2A phosphatase activity and cytokine expression. As shown in Fig. 9B, in cells where PP2A-C is overexpressed, OA is unable to inhibit PP2A activity to the same extent as controls. Further, cells transfected with PP2A-C plasmid significantly repressed OA-induced IL-6 and IL-8 mRNA expression (Fig. 9C,D) and protein secretion (Fig. 9E,F), respectively.

### OA inhibits TNFα-induced PP2A phosphatase activity and increases TNFα-induced IL-6 and IL-8 mRNA expression and protein secretion

**Figure 10:**
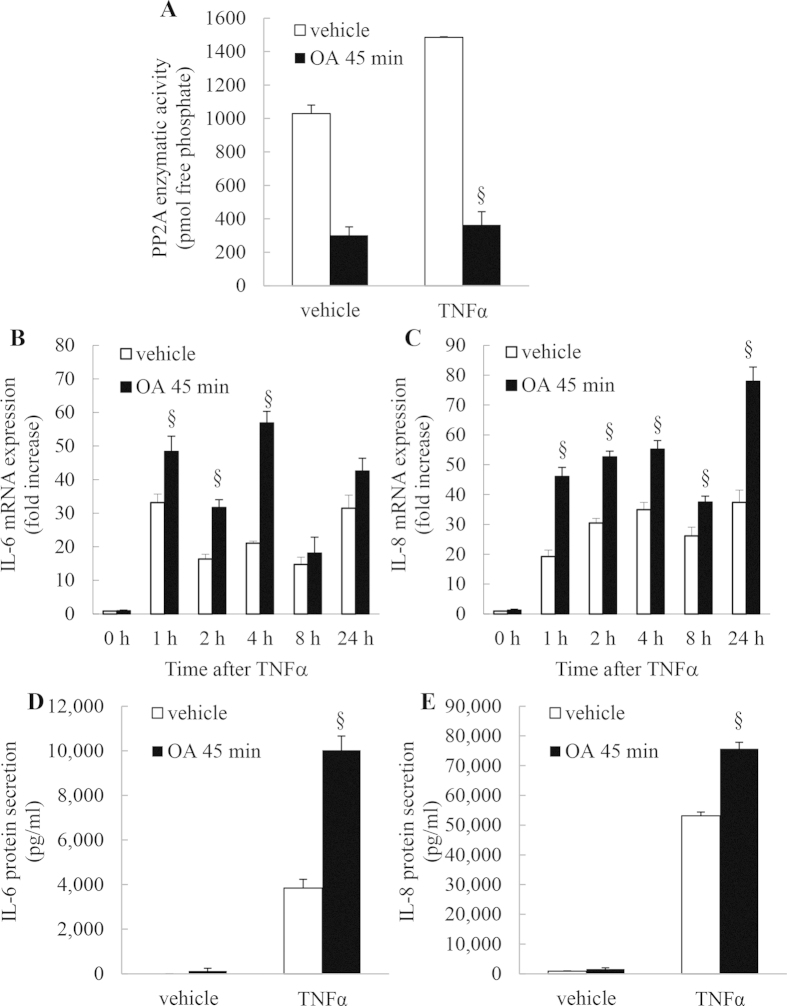
OA inhibits TNFα-induced PP2A phosphatase activity and increases TNFα-induced IL-6 and IL-8 mRNA expression and protein secretion. (**A**) PP2A enzymatic activity was measured in A549 cells treated for 45 min with vehicle or 1 μM OA. Cells were washed before stimulation with TNFα (4 ng/ml) and then PP2A enzymatic activity (measured as pmol free phosphate) was detected at 1 h. Statistical analysis was performed using one-way ANOVA then Bonferroni’s post-test (where § denotes a significant effect of OA on on TNFα-induced effects). (B-E) A549 cells were treated for 45 min with vehicle or 1 μM OA. Cells were washed before stimulation with TNFα (4 ng/ml). (**B**) IL-6 and (**C**) IL-8 mRNA expression was measured at 0, 1, 2, 4, 8, and 24 h (results expressed as fold increase compared to vehicle-treated cells at 0 h) and (**D**) IL-6 and (**E**) IL-8 protein secretion measured at 24 h. Statistical analysis was performed using two-way ANOVA then Bonferroni’s post-test (where §denotes a significant effect of OA on TNFα-induced effects (*P* < 0.05)). Data are mean ± SEM values from n = 3 independent experiments.

Our study thus far shows the important role played by basal PP2A enzymatic activity in restraining cytokine expression in unstimulated cells. To mimic the inflammatory milieu in asthma we now examined the impact of OA (for 45 min) on IL-6 and IL-8 expression stimulated by TNFα – a pro-inflammatory cytokine implicated in asthma. As shown in Fig. 10A, TNFα increases PP2A activity and this can be significantly repressed by OA. We then examined the time course of TNFα-induced IL-6 (Fig. 10B) and IL-8 (Fig. 10C) mRNA expression and found that OA treatment significantly increased TNFα-induced cytokine expression at a number of time-points and that this resulted in significant increase in TNFα-induced IL-6 and IL-8 secretion at 24 h (Fig. 10D,E, respectively: *P* < 0.05).

### Specific knockdown of PP2A with siRNA reduces TNFα-induced PP2A protein levels and activity and results in increased TNFα-induced IL-8 and IL-6 mRNA expression and protein secretion

**Figure 11:**
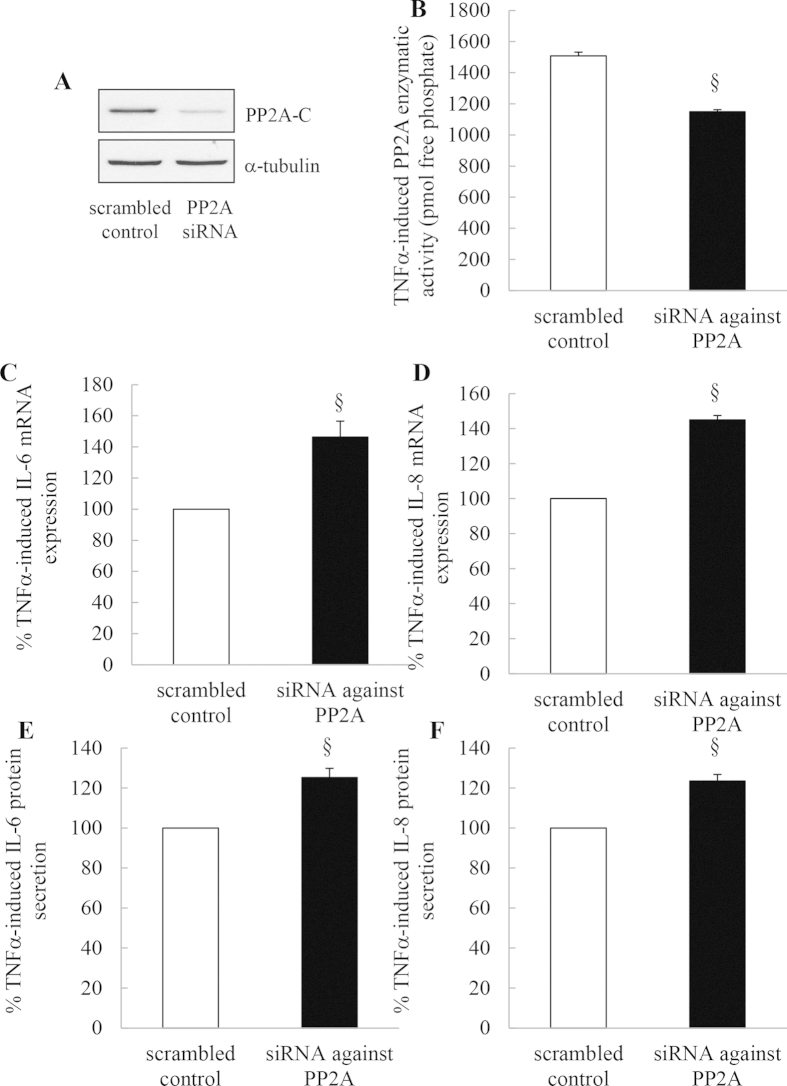
Specific knockdown of PP2A with siRNA reduces TNFα-induced PP2A protein levels and activity and results in increased TNFα-induced IL-8 and IL-6 mRNA expression and protein secretion. A549 cells transfected with scrambled control or siRNA against PP2A-C were stimulated with TNFα (4 ng/ml) before: (**A**) PP2A-C knockdown at 1 h was confirmed by Western blotting (representative results are shown as cropped blots and full-length blots are presented in Supplementary Figure 4); (**B**) PP2A enzymatic activity detected at 1 h (measured as pmol free phosphate); (**C**) IL-6 and (**D**) IL-8 mRNA expression measured at 1 h (results expressed as % of TNFα-induced mRNA expression in scrambled control (designated as 100%)) ; and (**E**, **F**) IL-6 and IL-8 protein secretion measured at 24 h (results expressed as % of TNFα-induced protein secretion in scrambled control (designated as 100%)). Statistical analysis was performed using Student’s unpaired t test (where § denotes a significant effect of siRNA against PP2A-C on TNFα-induced effects (*P* < 0.05)). Data are mean ± SEM values from n = 3 independent experiments.

Finally, to demonstrate that these OA-mediated effects were specific to PP2A, A549 cells were transfected with scrambled control or siRNA against PP2A-C before TNFα stimulation. When PP2A-C was knocked down (confirmed by Western blotting in Fig. 11A) we observed significant reduction in TNFα-induced PP2A enzymatic activity (Fig. 11B) with a corresponding upregulation of TNFα-induced IL-6 and IL-8 mRNA expression and protein secretion from A549 cells (Fig. 11C–F, respectively: *P* < 0.05)

## Discussion

To explore the role of PP2A in cytokine expression in human lung epithelial cells, we took multiple approaches to modulate PP2A phosphatase activity by using a PP2A inhibitor, OA; two PP2A activators, FTY720 and AAL(s); an expression plasmid to overexpress the catalytic subunit of PP2A (PP2A-C); and siRNA to knockdown PP2A-C. These studies reveal the important role played by PP2A in cytokine regulation in the context of airway inflammation and demonstrate the link between perturbation of PP2A activity and effects on functional outcomes such as cell signaling and cytokine expression.

Several studies have demonstrated that PP2A serves as a negative regulator of MAPKs^8^,^32^,^33^ and in support we found that inhibition of PP2A by OA upregulates p38 MAPK, ERK and JNK phosphorylation in A549 cells. These results demonstrate that the basal activity of PP2A in untreated cells restrains MAPK-mediated cell signaling in A549 cells. In our previous studies we demonstrated that IL-6 and IL-8 are two major cytokines upregulated via MAPK-mediated pathways^18^,^19^,^20^,^21^. JNK has been reported to be regulated by PP2A and play a role in IL-8 production in models of TNFα and lipopolysaccharide-induced lung inflammation, with an important consequences for severe asthma^34^,^35^.

Notably, PP2A inhibition also has a significant impact on the critical anti-inflammatory protein - TTP. TTP is an mRNA destabilizing protein that targets numerous cytokines^36^, including those involved in asthmatic inflammation. Its expression and function are p38 MAPK-dependent^26^,^27^. There is a dynamic equilibrium between unphosphorylated and phosphorylated forms of TTP. The unphosphorylated form of TTP is active and capable to decay cytokine mRNA. However, this form of TTP is unstable and undergoes proteosomal degradation, whereas phosphorylated TTP is stable but unable to target cytokines mRNA for decay. Importantly, TTP is a direct target of PP2A; as PP2A phosphatase activity is responsible for dephosphorylation of TTP at two key serines (Ser52 and Ser178)^25^. In this way, PP2A shifts this balance of TTP towards unphosphorylated TTP via dephosphorylation^25^,^27^. Our study demonstrates that inhibition of PP2A (with OA for 45 min) significantly induces TTP mRNA expression but the resulting TTP protein has electrophoretic mobility in immunoblots indicative of hyperphosphorylated forms of TTP. Our study concurs with previous reports that demonstrate that inhibition of PP2A phosphatase activity causes accumulation of hyperphosphorylated and stable TTP protein^25^,^37^, suggesting that in the absence of PP2A activity, the balance is shifted towards stable, but inactive, phosphorylated forms of TTP.

Our study utilizing OA demonstrates the important anti-inflammatory control exerted by PP2A phosphatase basal activity in A549 lung epithelial cells. These studies were confirmed in BEAS-2B cells. Given that OA is a non-selective pharmacological inhibitor, we have directly implicated PP2A with PP2A-C overexpression as well as PP2A activators. A number of small molecules have been reported to activate PP2A^4^. To date the best known of these is the sphingosine analog FTY720 (fingolimod) and we have utilized it herein overcome OA-mediated repression of PP2A enzymatic activity and repress cytokine expression. But it is important to note that FTY720 is also an agonist/antagonist of the sphingosine 1-phosphate (S1P) pathway and we and others have shown that S1P is pro-inflammatory and pro-asthmatic^29^,^30^,^31^,^38^,^39^. Therefore we utilized AAL(s), a PP2A activator devoid of S1P agonism^5^,^6^,^13^ to confirm the anti-inflammatory effect of basal PP2A in A549 cells.

Our study demonstrates that basal PP2A activity restrains cytokine expression in a cellular model of asthmatic inflammation and highlights an important role for MAPKs and TTP. Moreover, PP2A plays an important role in cytokine expression in cells stimulated with TNFα; we show that inhibition of PP2A with OA, and more specifically with PP2A-C knockdown by siRNA, results in significant increases in cytokine production. Taken together our study has revealed, in part, the molecular mechanisms of PP2A anti-inflammatory function and highlight the potential of boosting the power of endogenous phosphatases as novel anti-inflammatory strategies to combat asthmatic inflammation.

## Author Contributions

Conceived, designed and performed the experiments: M.M.R., N.N.R., A.J.A. Analysis and interpretation: A.R.C., N.M.V., A.J.A. Important intellectual content and reagents: J.C.M., A.R.C., N.M.V., A.J.A. Wrote the paper: M.M.R., A.J.A.

## Additional Information

**How to cite this article**: Rahman, M. M. *et al.* Basal protein phosphatase 2A activity restrains cytokine expression: role for MAPKs and tristetraprolin. *Sci. Rep.* **5,** 10063; doi: 10.1038/srep10063 (2015).

## Supplementary Material

Supplementary Information
