# Supplementary material for: Basal protein phosphatase 2A activity restrains cytokine expression: role for MAPKs and tristetraprolin
Source: Sci Rep. 2015 May 18;5:10063. doi: 10.1038/srep10063 (PMC4434956; doi:10.1038/srep10063)
Supplement: Supplementary Information [file srep10063-s1.pdf]

# Basal protein phosphatase 2A activity restrains cytokine expression: role for MAPKs and tristetraprolin

Md. Mostafizur Rahman, Nowshin N. Rumzhum, Jonathan C. Morris, Andrew R. Clark, Nicole M. Verrills and Alaina J. Ammit

# phospho-p38 MAPK

0 h    0.5 h    1 h

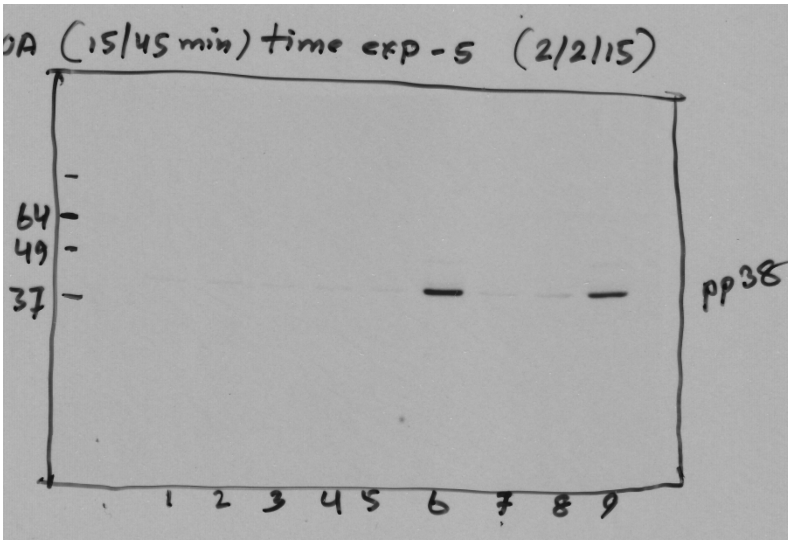


| | | | | | | | | | |
|---|---|---|---|---|---|---|---|---|---|
| OA 15 min | - | + | - | - | + | - | - | + | - |
| OA 45 min | - | - | + | - | - | + | - | - | + |

# p38 MAPK

0 h    0.5 h    1 h

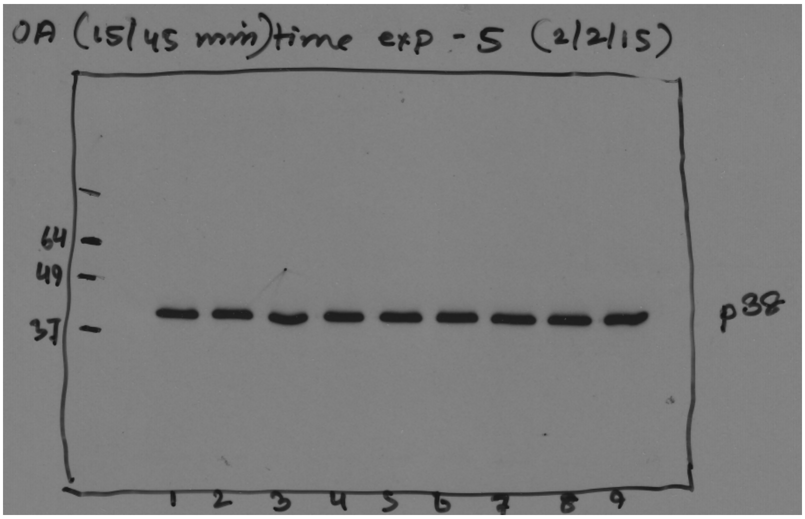


| | | | | | | | | | |
|---|---|---|---|---|---|---|---|---|---|
| OA 15 min | - | + | - | - | + | - | - | + | - |
| OA 45 min | - | - | + | - | - | + | - | - | + |

**Supplementary Figure 1A**

# phospho-ERK

0 h | 0.5 h | 1 h

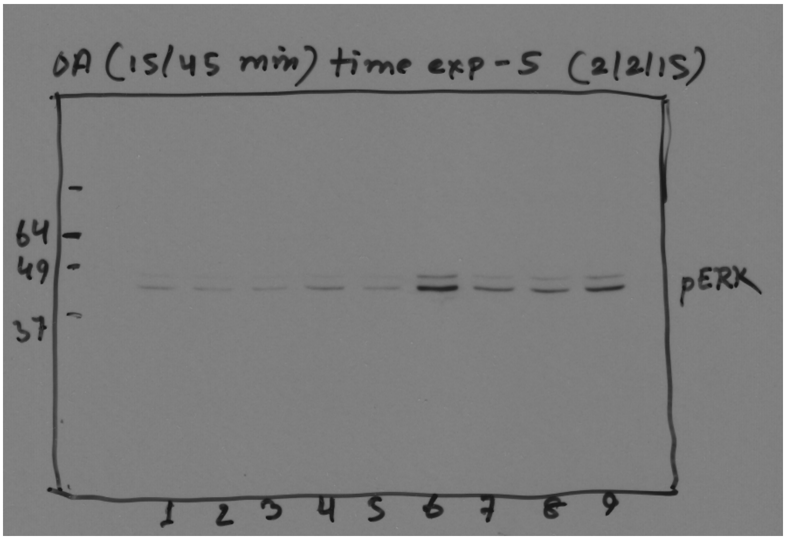


| | | | | | | | | | |
|---|---|---|---|---|---|---|---|---|---|
| OA 15 min | - | + | - | - | + | - | - | + | - |
| OA 45 min | - | - | + | - | - | + | - | - | + |

# ERK

0 h | 0.5 h | 1 h

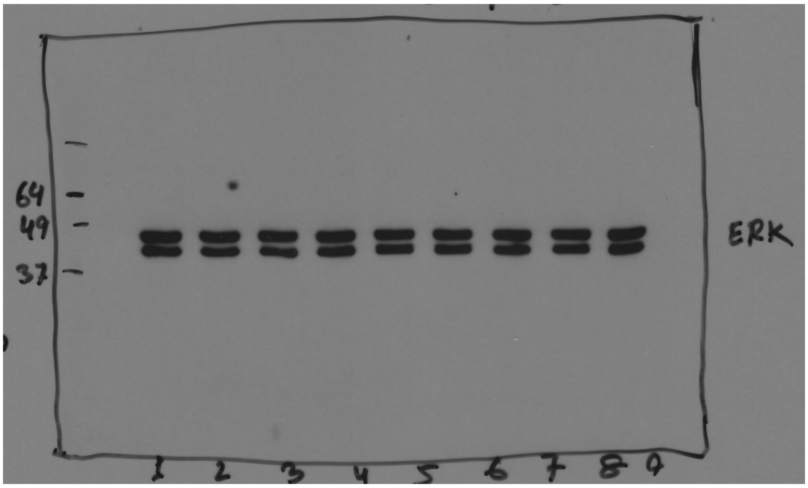


| | | | | | | | | | |
|---|---|---|---|---|---|---|---|---|---|
| OA 15 min | - | + | - | - | + | - | - | + | - |
| OA 45 min | - | - | + | - | - | + | - | - | + |

**Supplementary Figure 1B**

# phospho-JNK

0 h　　0.5 h　　1 h

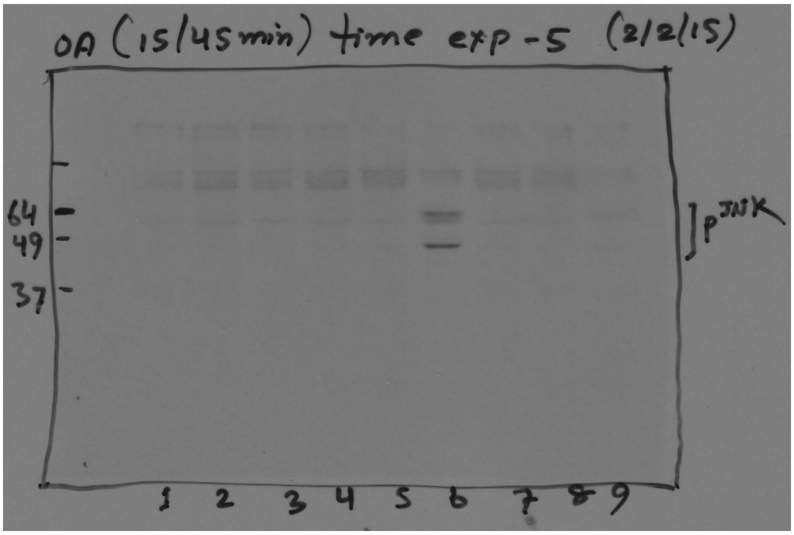


| | | | | | | | | | |
|---|---|---|---|---|---|---|---|---|---|
| OA 15 min | - | + | - | - | + | - | - | + | - |
| OA 45 min | - | - | + | - | - | + | - | - | + |

# JNK

0 h　　0.5 h　　1 h

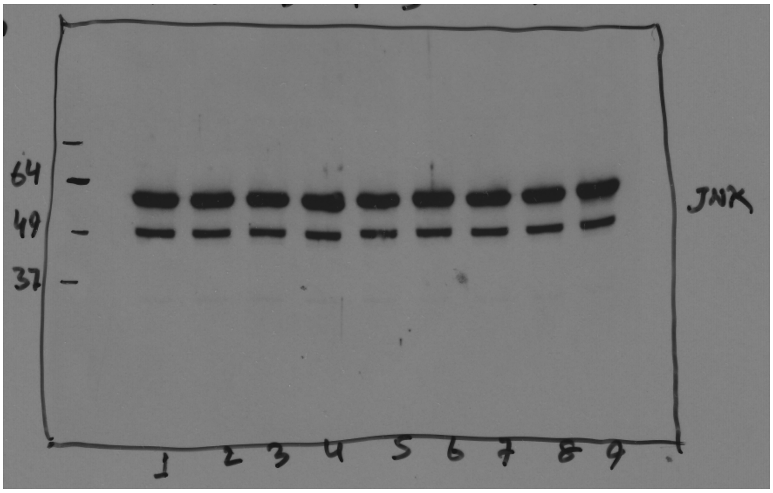


| | | | | | | | | | |
|---|---|---|---|---|---|---|---|---|---|
| OA 15 min | - | + | - | - | + | - | - | + | - |
| OA 45 min | - | - | + | - | - | + | - | - | + |

**Supplementary Figure 1C**

**TTP**

0 h 0.5 h 1 h

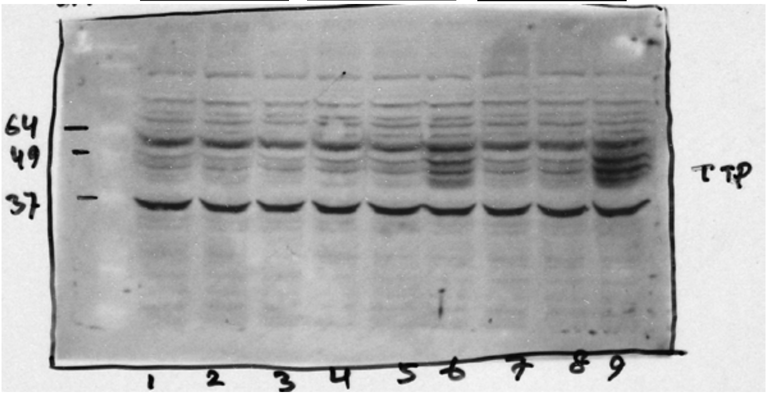


| | | | | | | | | | |
|---|---|---|---|---|---|---|---|---|---|
| OA 15 min | - | + | - | - | + | - | - | + | - |
| OA 45 min | - | - | + | - | - | + | - | - | + |

**α-tubulin**

0 h 0.5 h 1 h

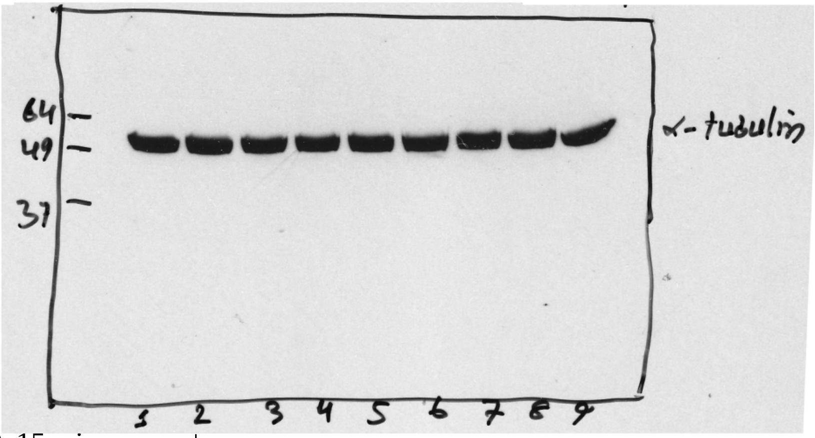


| | | | | | | | | | |
|---|---|---|---|---|---|---|---|---|---|
| OA 15 min | - | + | - | - | + | - | - | + | - |
| OA 45 min | - | - | + | - | - | + | - | - | + |

**Supplementary Figure 2**

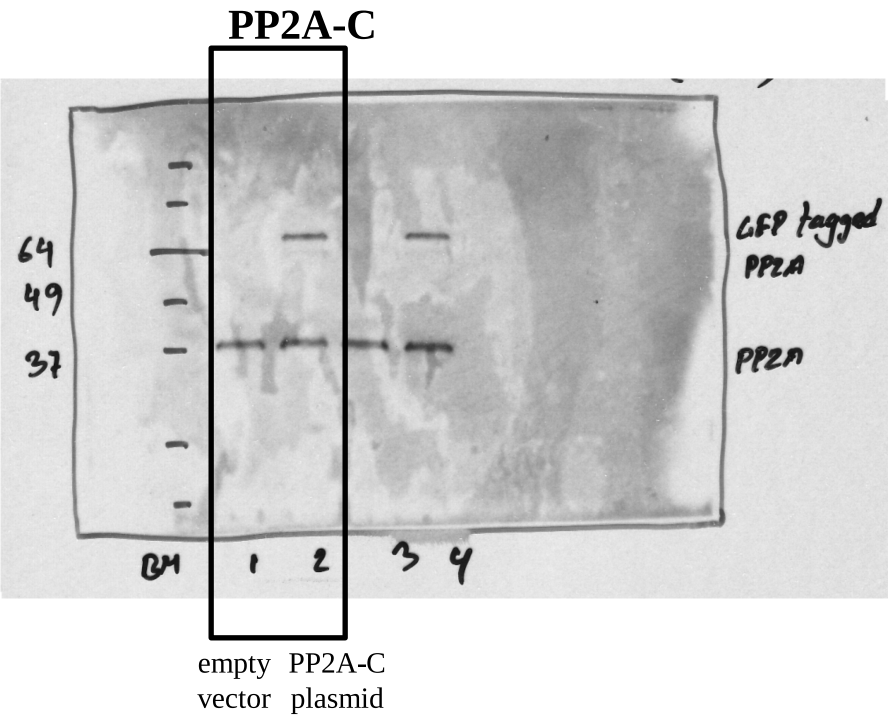


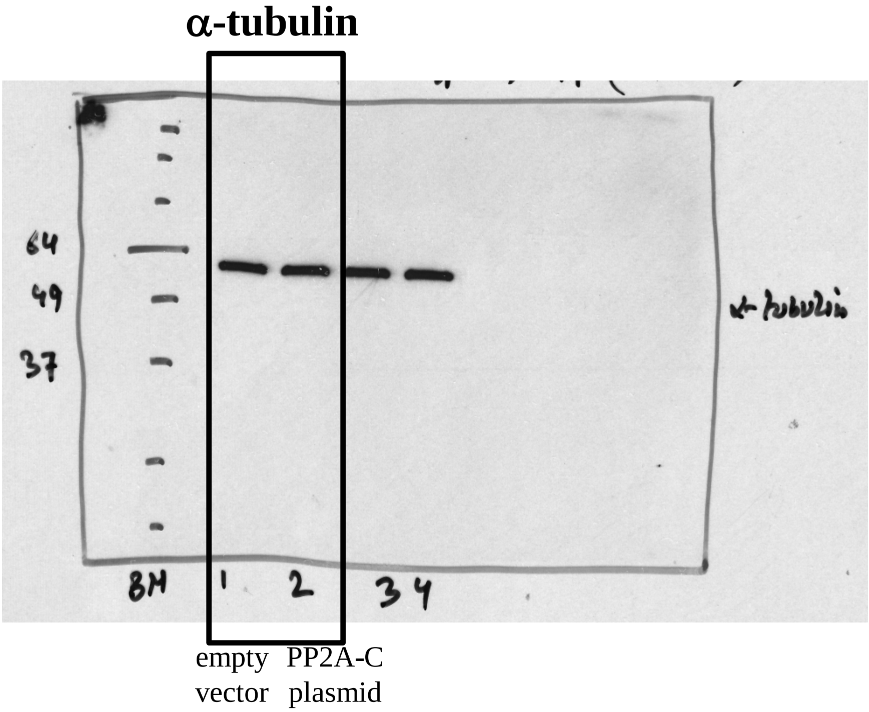


**Supplementary Figure 3**

## siRNA against PP2A

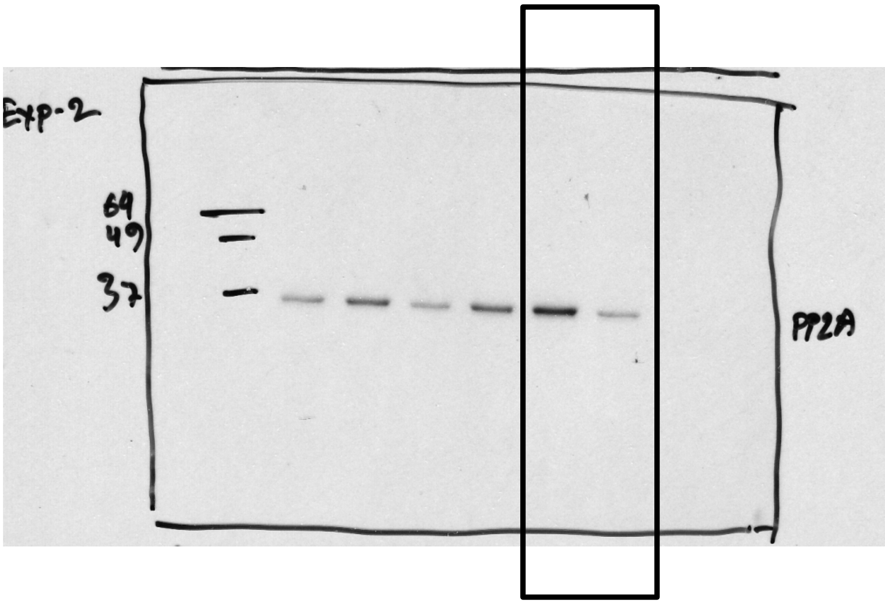


scrambled control PP2A siRNA

## α-tubulin

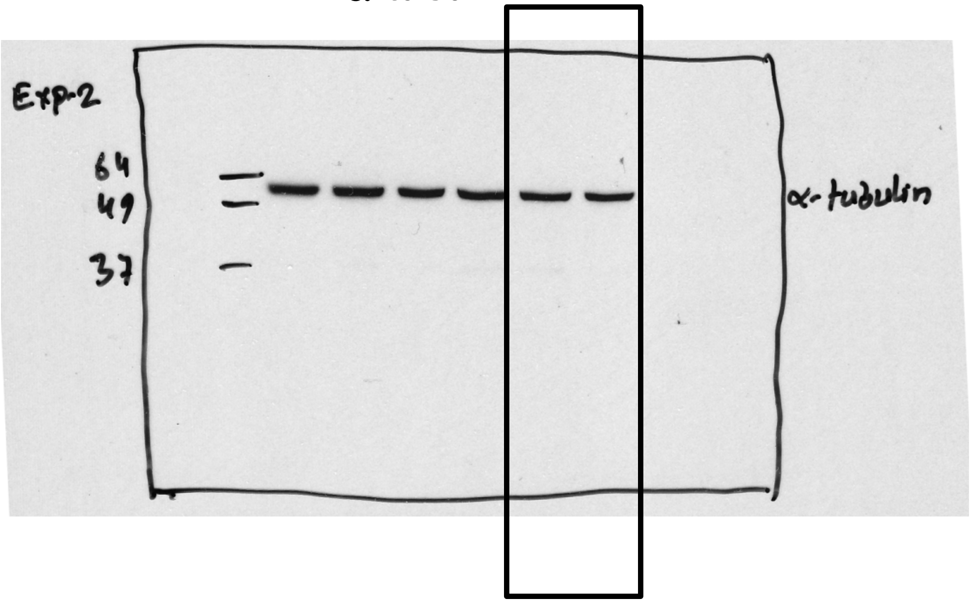


scrambled control PP2A siRNA

**Supplementary Figure 4**
